# Cross-Regional Reanalysis of 16S rDNA Amplicon Sequence Data Reveals Core Taxa and Regional Modular Dynamics in the *Panax ginseng* Rhizosphere Bacterial Community

**DOI:** 10.3390/plants15213473

**Published:** 2026-11-06

**Authors:** Md Minarul Islam, Yunseon Hwang, Yongsoo Choi, Ki Sung Kang

**Affiliations:** 1 Department of Food Science and Biotechnology, Gachon University, Seongnam 13120, Republic of Korea; minarul2026@gachon.ac.kr; 2 College of Korean Medicine, Gachon University, Seongnam 13120, Republic of Korea; pes1020@gachon.ac.kr; 3 Center for Natural Product Efficacy Optimization, Korea Institute of Science and Technology (KIST), Gangneung 25451, Republic of Korea; 4 Natural Product Applied Science, KIST School, University of Science and Technology, Daejeon 34113, Republic of Korea

**Keywords:** rhizosphere, *Panax ginseng*, microbiome, 16S rDNA, BioProjects

## Abstract

The rhizosphere microbiome plays a crucial role in the development, health, and therapeutic properties of *Panax ginseng*. Therefore, we evaluated the spatial trends in bacterial communities using a comprehensive reanalysis of 359 sequenced rhizosphere samples from seven NCBI Sequence Read Archive BioProjects. The SILVA reference database was used to compare the Chinese and Korean sequencing datasets, focusing on quality filtering, denoising, amplicon sequence variants, and taxonomy assignment. The bacterial community structure was characterized, and differentially abundant taxa were identified using the Kruskal–Wallis test with false discovery rate correction. In addition, based on the detected bacterial taxa, potential ecological traits were contextually interpreted to provide insight into their potential ecological significance. Despite regional and technical variations, 37 bacterial taxa consistently emerged as conserved core microbiome members. Regional analyses revealed distinct bacterial drivers, such as *Ligilactobacillus*, *Romboutsia*, *Nitrospira*, and *Bradyrhizobium* in the Korean samples and *Holophaga* and Arachidicoccus in the Chinese samples. Putative functional annotations showed that plant-associated defense mechanisms, nutrient mobilization, and carbon cycling varied geographically. Overall, these findings indicate that although a conserved core community exists, regional differences may influence the composition, functional potential, and ecological relationships of the *P. ginseng* rhizosphere.

## 1. Introduction

The rhizosphere, a thin layer of soil surrounding plant roots, is influenced by root exudates and is one of the most biologically vibrant interfaces on Earth. The rhizosphere is a dynamic hub for interactions between plants and soil microorganisms, serving vital functions in plant nutrition, stress resilience, and productivity [1]. *Panax ginseng*, a medicinal herb of the *Araliaceae* family, is widely recognized for its therapeutic potential. The major bioactive components of ginseng, termed ginsenosides, are credited with broad pharmacological effects, including anti-inflammatory, anticancer, antimicrobial, antiviral, immunomodulatory, antidiabetic, and antioxidant properties [2,3]. In perennial medicinal plants, such as *P. ginseng Meyer*, which require years of field cultivation to reach therapeutic maturity, the rhizosphere microbiome is often recognized as the plant’s second genome, serving more than just a collection of passive inhabitants [4,5]. These complex microbial communities play a crucial role in plant growth and ginsenoside synthesis by facilitating nutrient cycling, regulating phytohormone signaling, enhancing plant immunity, and suppressing the growth of pathogenic microbes. The composition and function of rhizosphere microbes are influenced by both plant genotypes and environmental factors [6,7]. However, geographical variation remains a significant factor affecting the assembly and diversity of microbial communities in various cultivated areas. Moreover, variations in the hypervariable regions of 16S rRNA can introduce methodological biases, complicating comparisons across analyses and ecological insights [8,9,10]. While numerous earlier studies have detailed the rhizosphere microbial communities of *P. ginseng* in specific cultivation areas [11,12,13,14], it remains unclear which bacterial taxa are potentially linked to the *P. ginseng* rhizosphere across different geographical locations and which components of community composition differ regionally. Specifically, the degree to which a common core bacterial community exists alongside region-specific microbial patterns has not been rigorously investigated across distinct datasets.

Advances in next-generation sequencing have revolutionized microbiome research by enabling the study of both culturable and non-culturable microbes. Coupled with the increasing accessibility of public sequencing datasets, these technologies enable researchers to explore microbiome composition, assembly processes, and ecological patterns across various geographical and environmental contexts [15,16]. The use of 16S rRNA gene amplicon sequencing has become fundamental in microbiome research, as it enables rapid, cost-effective, and culture-independent simultaneous identification and assessment of bacterial community composition and relative abundance across many samples [17]. Next-generation sequencing (NGS) serves as a molecular fingerprint for taxonomic identification, and the high efficiency of NGS platforms has facilitated high-throughput data generation, making large-sample studies more feasible and affordable [18]. Most studies utilize it to create taxonomic profiles from individual experiments, primarily focusing on the microorganisms present and their relative abundances. However, the integrated reanalysis of microbiome datasets from multiple independent studies enables researchers to obtain more robust, generalizable, and biologically relevant insights than from individual studies alone. Combining datasets from different groups, locations, and experiments enhances the statistical power and allows for the identification of shared microbial patterns. However, this integration presents challenges, as datasets often differ in sampling techniques, sequencing methods, and study designs, leading to batch effects, heterogeneity, compositionality, and zero-inflation issues that require careful management through appropriate data harmonization, transformation, and batch-correction strategies [19,20,21]. Integrated reanalysis of microbiome data could offer broader insights into microbial community composition, ecological associations, and community structure. The integration of various data types and analytical methods offers a holistic perspective on microbial community composition, metabolic activities, and ecological relationships [22,23]. This approach integrates nonparametric differential abundance testing, the literature-supported ecological characterization of microbial taxa, and co-occurrence network analysis to describe taxonomic shifts, anticipated ecological roles, and community interaction patterns [24,25].

In this study, 359 sequenced samples of ginseng rhizosphere soil were retrieved from the NCBI Sequence Read Archive (SRA) across seven BioProject accessions to investigate geographic variation in the ginseng rhizosphere microbiome community. We aim to assess and compare the ecological profiles depicted by the V3–V4 Chinese group and V4 Korean cohort sequencing datasets. By identifying shared and region-associated taxa, we characterized regional variation in the *P. ginseng* rhizosphere bacterial community. These findings highlight the potential of integrating publicly available 16S rRNA gene amplicon datasets to reveal core and regional ecological patterns in plant–soil systems.

## 2. Results

### 2.1. Sequencing Depth and Data Quality

**Figure 1:**
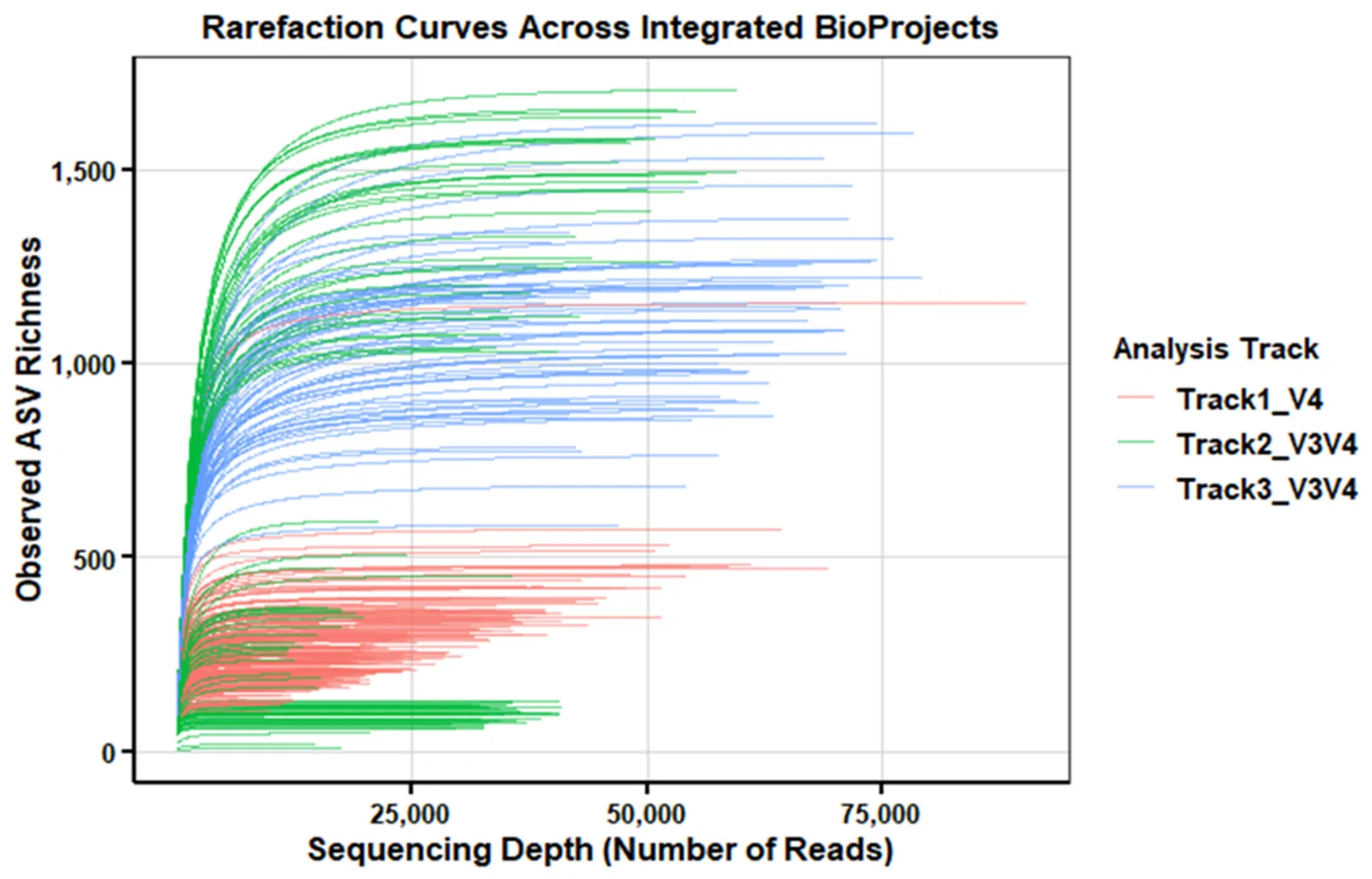
Rarefaction curves for the integrated BioProjects. Track 1 corresponds to the V4 dataset (206 samples), Track 2 corresponds to the V3–V4 dataset (90 samples), and Track 3 corresponds to the V3–V4 dataset (63 samples). Each curve represents a separate sample, illustrating observed ASV richness as a function of sequencing depth.

For each of the 359 samples, rarefaction analysis was used to assess the correlation between observed ASV richness and sequencing depth. The three sequencing tracks had different median sequencing depths: 20,939, 33,782, and 59,597 reads for Track 1, Track 2, and Track 3, respectively. Sequencing depth varied from 7567 to 90,279 reads across all samples, with a median of 29,977 reads. In general, ASV richness increased with sequencing depth on rarefaction curves; at higher sequencing depths, several curves showed a decreased slope (Figure 1). Across seven BioProjects, read retention was calculated at the BioProject level. Final non-chimeric read retention varied from 69.38% to 90.51%, while filtering retention varied from 83.98% to 98.31%. Four BioProjects had their chimeric reads eliminated: 7168 reads from PRJNA681095, 194,912 from PRJNA707387, 83,339 from PRJNA795020, and 27,635 from PRJNA927237. PRJNA1191688, PRJNA701796, and PRJNA704771 did not have any chimeric reads eliminated. Supplementary Table S1 contains comprehensive raw, filtered, denoised, and final non-chimeric read counts.

### 2.2. Micro-Regional Homeostasis of Rhizosphere Alpha Complexity Baselines

**Figure 2:**
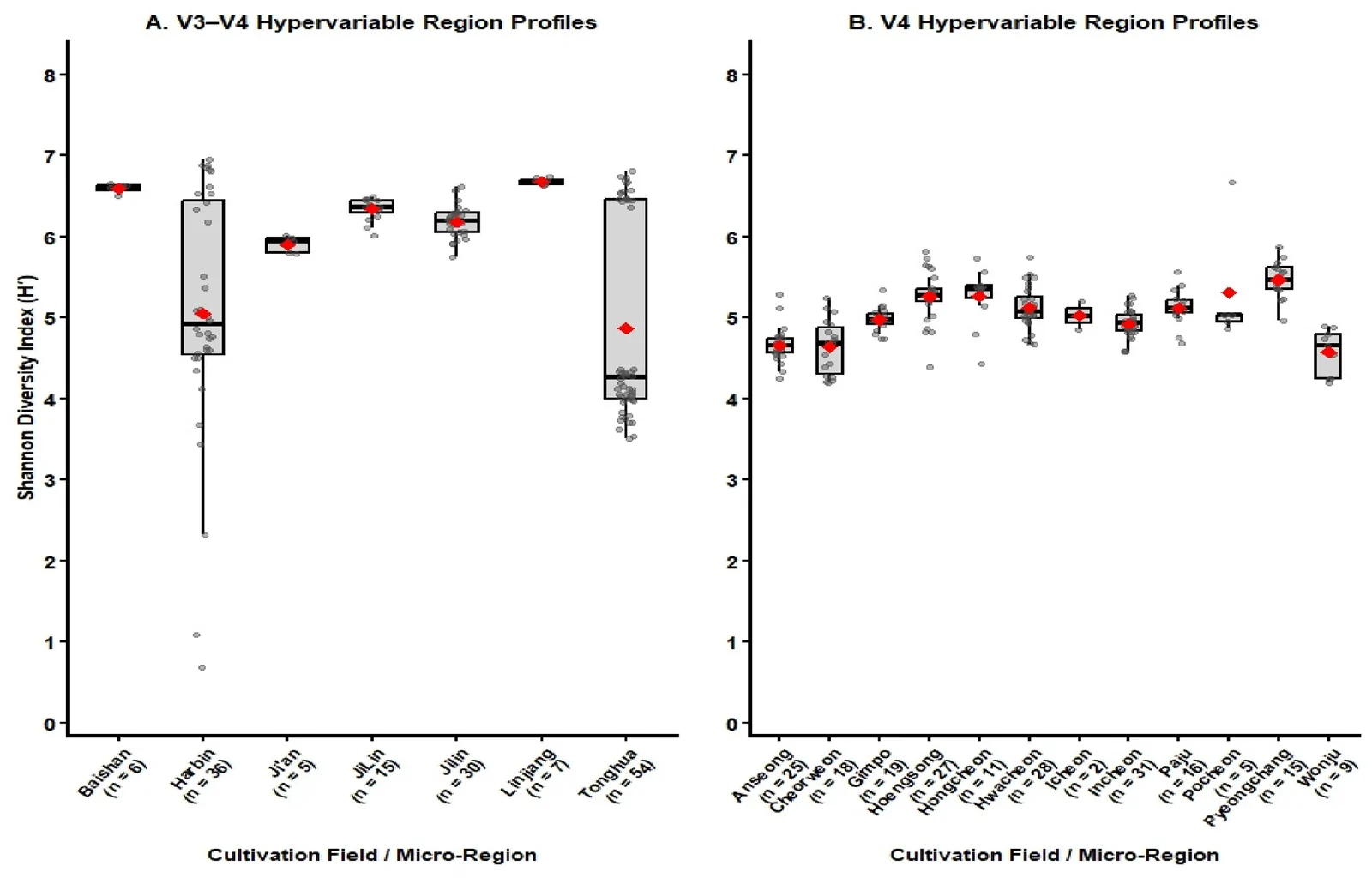
Regional patterns of within-sample bacterial alpha diversity. Shannon diversity indices were measured for (**A**) V3–V4 profiles from Chinese regional groups and (**B**) V4 profiles from South Korean regional groups. Boxplots display medians, interquartile ranges, and overall ranges, with individual data points signifying SRA samples. Regional variations were evaluated using the Kruskal–Wallis test (*p* < 0.001).

To determine whether geographic variations in agricultural practices and local soil characteristics affect the ecological complexity of the *P. ginseng* rhizosphere microbiome, alpha diversity patterns were examined across different cultivation regions (Figure 2). In the Chinese V3–V4 sequencing dataset (Figure 2A), the rhizosphere communities maintained relatively consistent diversity levels, with median Shannon diversity indices typically ranging from approximately 5.8–6.5 in Baishan, Ji’an, Jilin, and Linjiang. The Harbin cohort exhibited greater variability, as evident from a wider interquartile range and a lower median diversity (H′ ≈ 4.9), whereas the Tonghua samples had the lowest median diversity (H′ ≈ 4.3). Although substantial overlap was observed among several cultivation sites, Shannon diversity varied significantly among the Chinese geographical groups (Kruskal–Wallis χ^2^ = 38.845, df = 6, *p* = 7.68 × 10^−7^). Dunn’s post hoc pairwise comparisons utilizing the Benjamini–Hochberg adjustment revealed notable differences between Baishan and Harbin, Baishan and Ji’an, Baishan and Tonghua, Harbin and Linjiang, Ji’an and Linjiang, Jilin and Linjiang, Jilin and Tonghua, as well as Linjiang and Tonghua (BH-adjusted *p* < 0.05).

A similar trend was observed in the Korean V4 sequencing track (Figure 2B). Samples from 11 different cultivation regions throughout the Korean Peninsula exhibited relatively consistent rhizosphere microbial diversity. Median Shannon diversity values generally ranged from 4.5 to 5.5 across Anseong, Cheorwon, Gimpo, Hoengseong, Hongcheon, Hwacheon, Icheon, Incheon, Paju, Pocheon, and Pyeongchang, with only the Wonju group exhibiting slightly lower diversity values. However, there were statistically significant overall differences among Korean geographic groupings (Kruskal–Wallis χ^2^ = 108.308, df = 11, *p* = 3.99 × 10^−11^). In 32 out of 66 geographic comparisons, significant differences were found using Dunn’s pairwise comparisons with Benjamini–Hochberg correction (BH-adjusted *p* < 0.05). Thus, the statistical analyses revealed significant geographic variance in alpha diversity in both the Chinese V3–V4 and Korean V4 datasets, despite the fact that Shannon diversity distributions overlapped significantly across cultivation regions.

### 2.3. National and Micro-Regional Beta Diversity Dispersal Patterns

**Figure 3:**
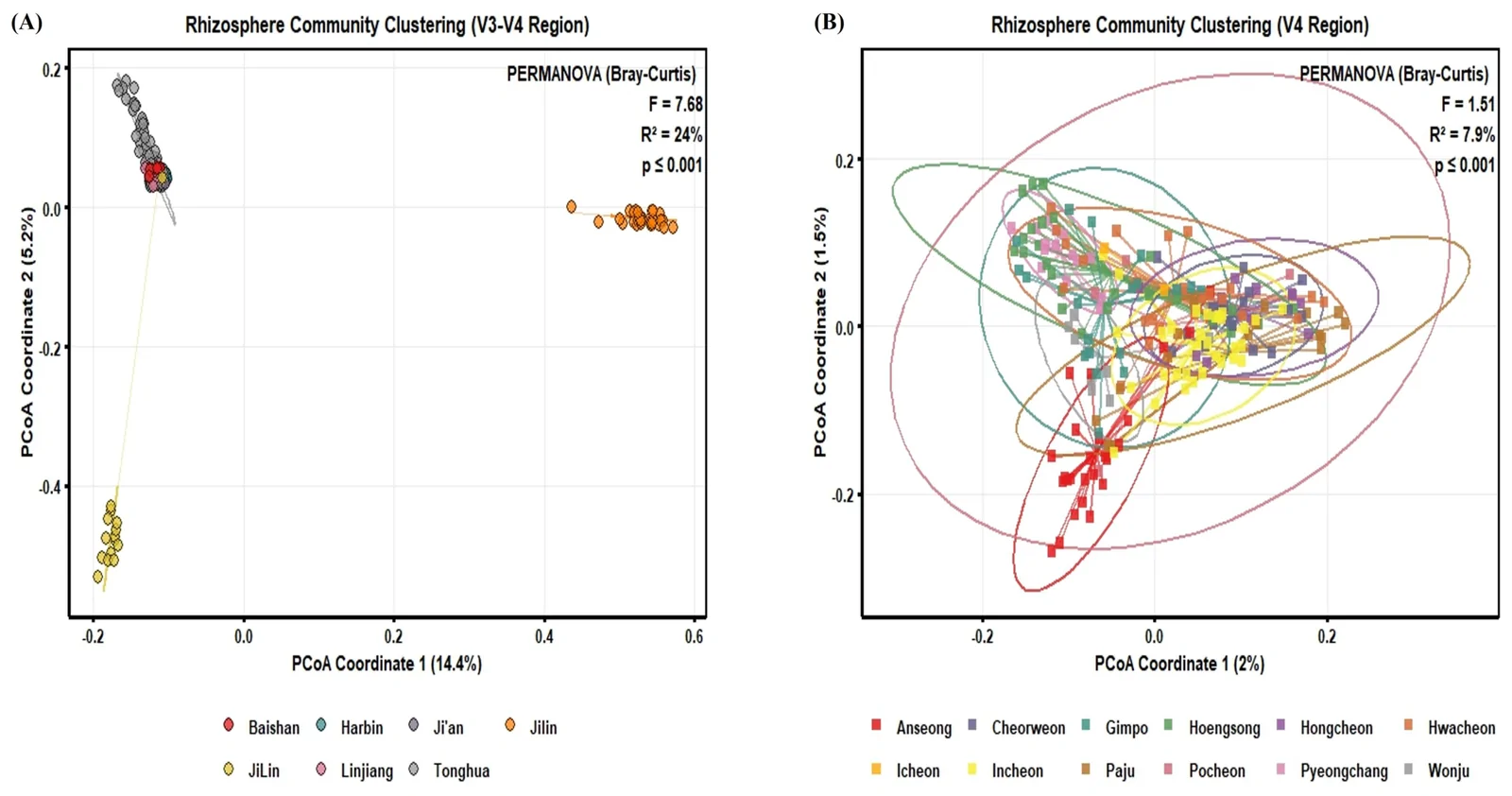
Ordination of beta diversity and community clustering among regional cohorts. Principal coordinate analysis (PCoA) utilizing Bray–Curtis dissimilarity matrices for (**A**) the combined V3–V4 hypervariable region track (Tracks 2 and 3) and (**B**) the V4 hypervariable region track (Track 1). Ellipses denote 95% confidence intervals encircling micro-regional centroids. Statistical differentiation among regional cultivation areas was assessed through permutational multivariate analysis of variance (PERMANOVA; 999 permutations), incorporating pseudo-F statistics, effect sizes (R^2^), and significance levels (*p*-value) noted within each panel.

The ordination of community profiles revealed distinctly different ecological assembly patterns between the two national profiling streams (Figure 3). In the processing stream focusing on the V3–V4 hypervariable region, which depicted the Chinese rhizosphere cohorts (Figure 3A), micro-regional geographic origin had a significant and polarized impact on the overall taxonomic assembly (F = 7.68, R^2^ = 24%, *p* < 0.001), dividing the profiles into distinct, isolated ecological areas. Samples from the Jilin area were completely separated along the positive axis of PCoA Coordinate 1, whereas there was a strong multi-study consensus among the Tonghua, Harbin, Ji’an, Baishan, and Linjiang groups, which clustered together in the upper-left quadrant.

Remarkably, the pronounced geographic fragmentation observed in Chinese fields was completely eclipsed by a well-maintained structural baseline within the V4 hypervariable region dataset, which aligned with the Korean cultivation pathways across multiple cities (Figure 3B). The PERMANOVA analysis of the V4 Korean matrix revealed that geographic location accounted for 7.9% of the overall compositional variance (R^2^ = 7.9%, F = 1.51, *p* < 0.001). Rather than being divided into distinct areas based on local coordinates, the confidence ellipses for Korean regions, such as Anseong, Hoengsong, Gimpo, Hwacheon, and Paju, exhibited spatial overlap, indicating a remarkably consistent nationwide homeostatic community structure across the Korean Peninsula.

### 2.4. Taxonomic Architecture of the Conserved Core Rhizosphere Microbiome

Intensive analysis of cross-regional datasets revealed a unique core microbiome comprising 37 bacterial taxa that were reliably identified in *P. ginseng* rhizosphere samples from various geographic areas, even with variations in national origin and technical batch (Supplementary Table S2). Rather than being randomly and arbitrarily distributed across a wide array of soil microflora, these 37 core taxa exhibited a well-organized, conservative taxonomic structure, primarily within the phyla Acidobacteriota, Proteobacteria, Actinobacteriota, and Gemmatimonadota (Supplementary Table S2).

A comparative evaluation of the two independent processing streams revealed remarkable evolutionary consistency across analyses. The actinobacterial genus *Gaiella* effectively transcended technical, experimental, and geopolitical boundaries, establishing itself as a stable core member in both the longer V3–V4 Chinese profiling dataset (1.828% mean abundance) and the shorter V4 Korean pipeline (1.160% mean abundance). However, the rest of the architecture is characterized by distinct regional patterns. In the V4 Korean sequencing track, the root-associated structure was significantly supported by unclassified genera from the phylum Acidobacteriota, with peak mean global relative abundances of 4.985% and 3.24%, respectively, along with a strong baseline for Gemmatimonadota (3.089%). Plant-associated mutualists established a significant dominant presence in the Korean matrix, led by *Sphingomonas* (1.895%), *Pseudolabrys* (1.909%), and *Acidibacter* (1.247%).

### 2.5. Taxonomic Structure of the Ginseng Rhizosphere Bacterial Community

**Figure 4:**
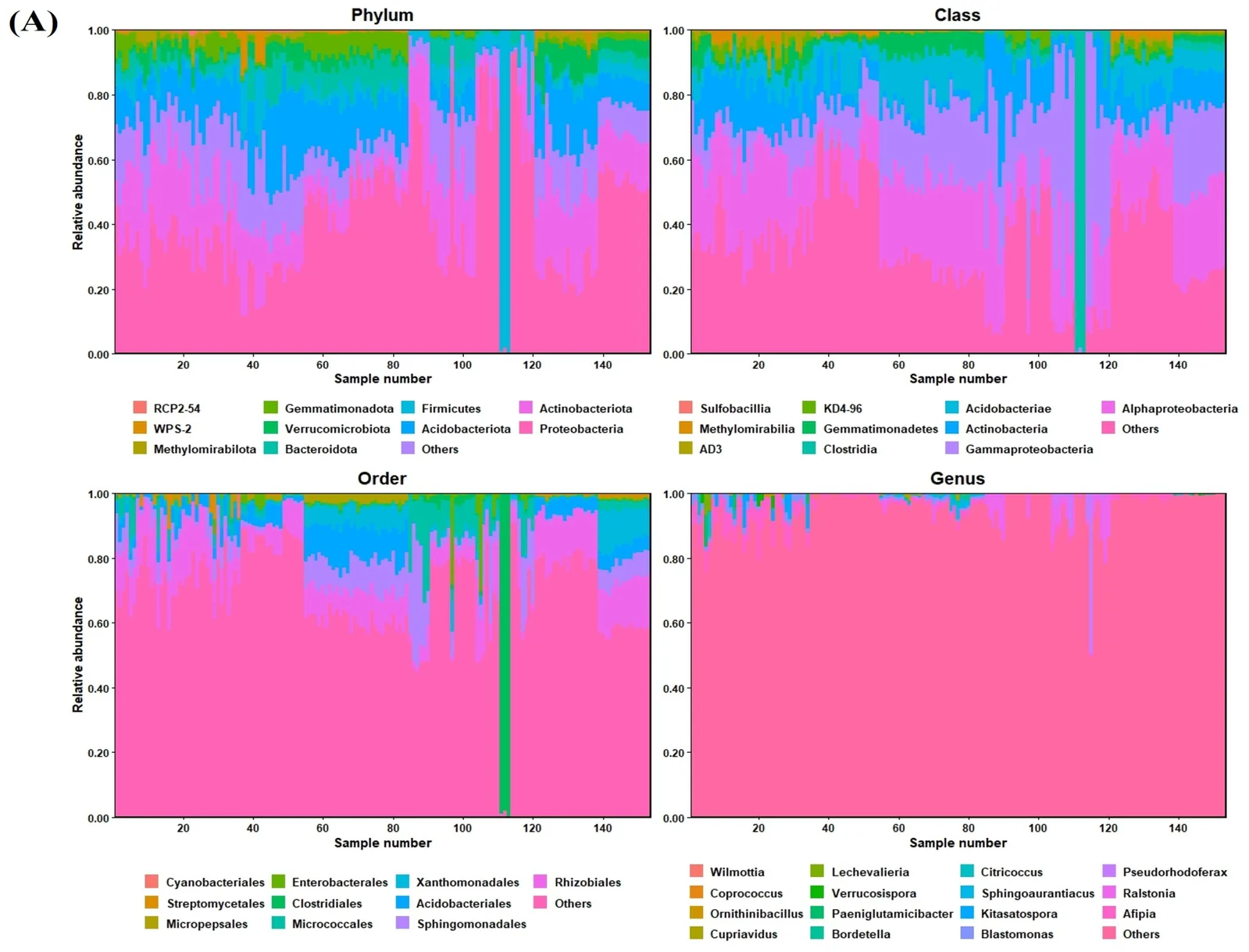

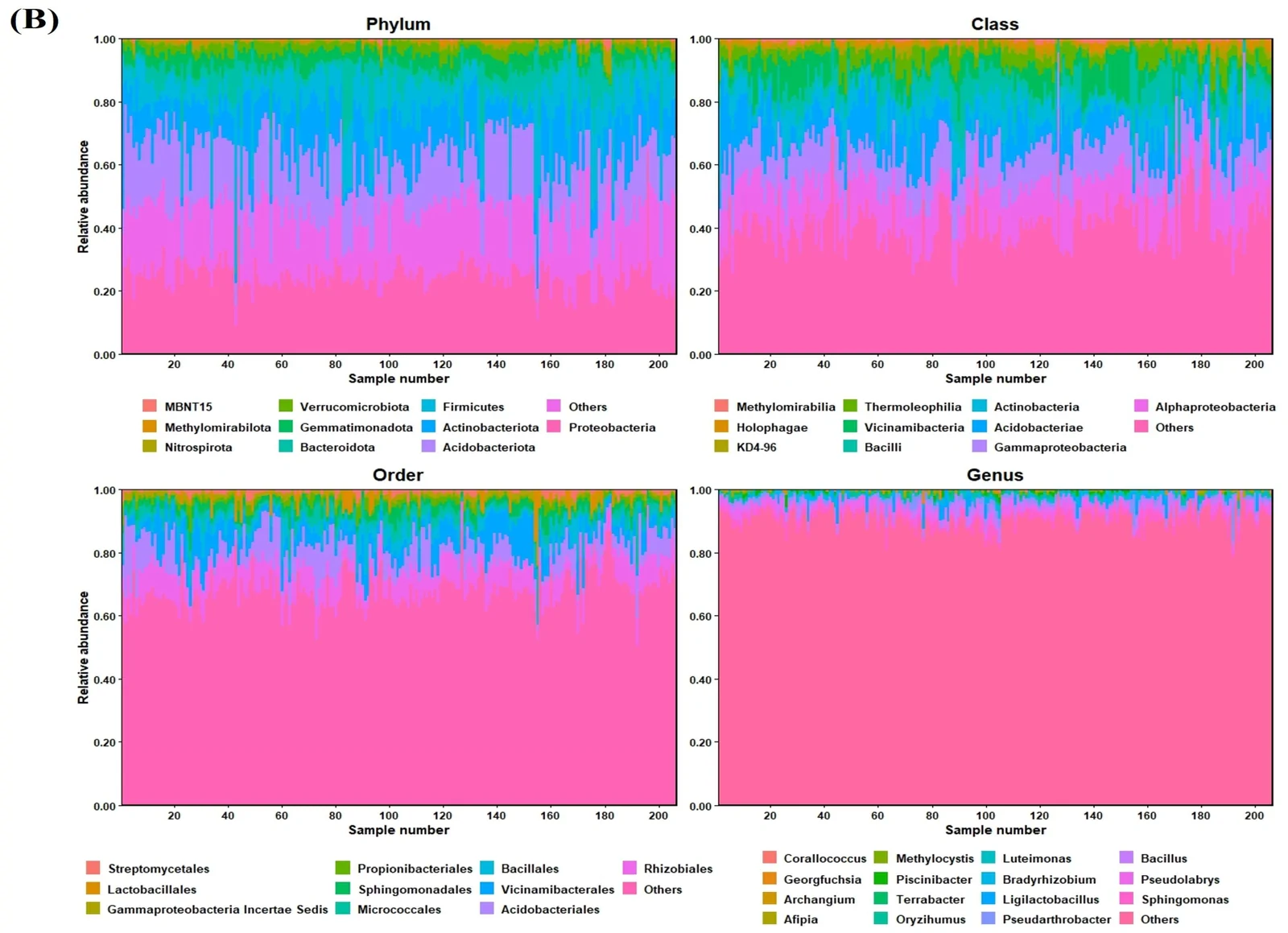
Taxonomic structure of bacterial communities in the ginseng rhizosphere at the phylum, class, order, and genus levels. (**A**) Chinese V3–V4 dataset; (**B**) Korean V4 dataset. Relative abundances of predominant taxa are shown.

For the Chinese V3–V4 and Korean V4 datasets, the bacterial taxonomic composition was assessed distinctly at the phylum, class, order, and genus levels (Figure 4). Proteobacteria and Actinobacteriota were the two most common phyla in the Chinese dataset (Figure 4A), with the considerable presence of Bacteroidota, Firmicutes, Acidobacteriota, and Verrucomicrobiota. Rhizobiales constituted a significant portion at the order level, whereas Alphaproteobacteria and Gammaproteobacteria were prominent at the class level. The most common phyla in the Korean dataset (Figure 4B) were Proteobacteria, Actinobacteriota, Acidobacteriota, Firmicutes, and Bacteroidota; at the class level, Alphaproteobacteria and Gammaproteobacteria were also present. Sphingomonadales, Burkholderiales, Micrococcales, and Rhizobiales were among the other orders that made major contributions to the observed patterns. Both datasets showed a more dispersed taxonomic structure at the genus level, with many genera contributing to the community and no single genus continuously predominating across samples. Overall, both datasets shared a number of major taxonomic categories; however, the relative abundance profiles of specific taxa differed between samples and datasets.

### 2.6. Regional Niche Differentiation Is Driven by Distinct Taxonomic Modulators

**Figure 5:**
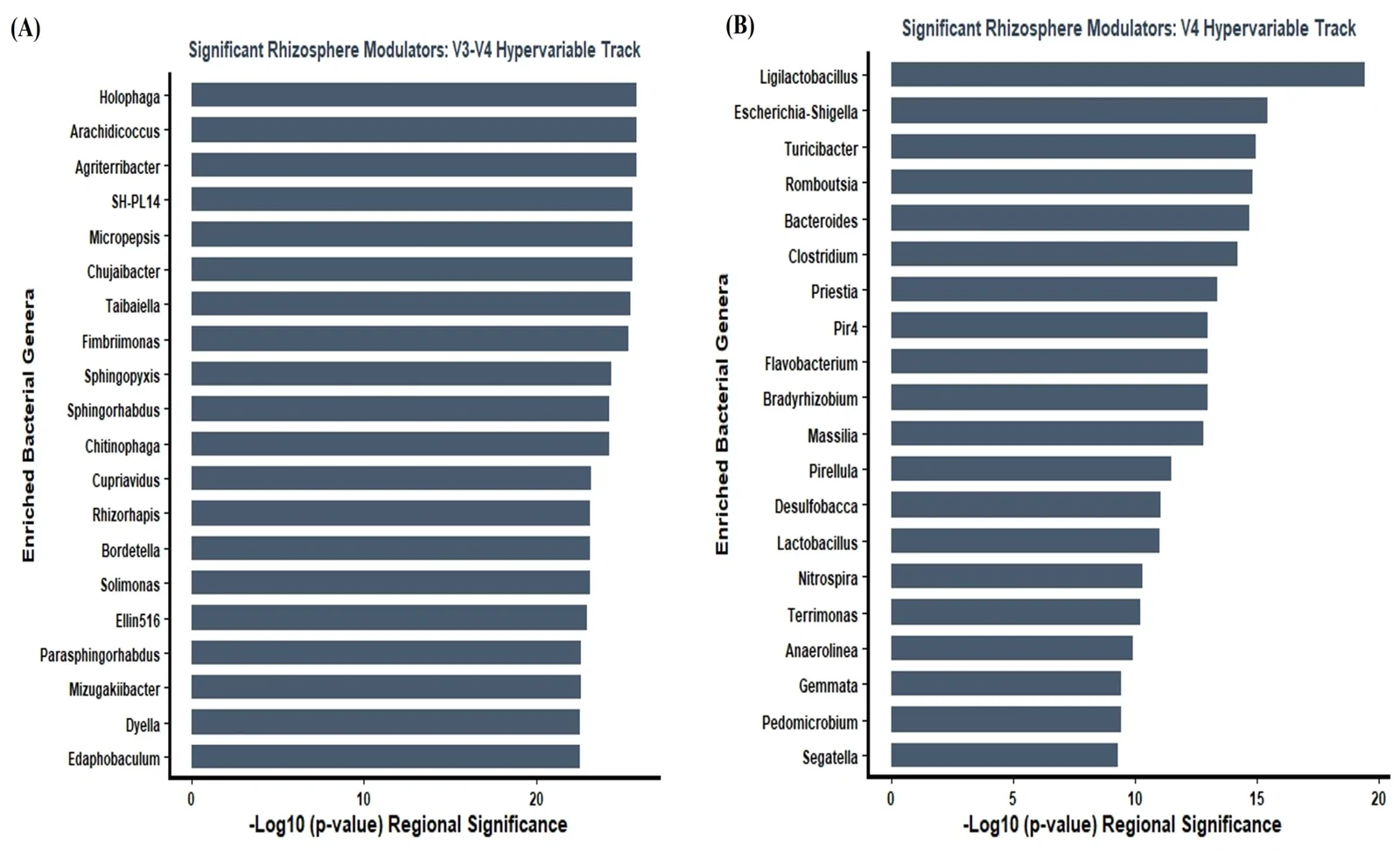
Major enriched bacterial genera related to regional niche variation in the *Panax ginseng* rhizosphere. Differentially abundant genera among geographic regions were detected using the non-parametric Kruskal–Wallis test (*p* < 0.05). The top 20 genera were arranged based on −log_10_(*p*-value) for (**A**) the integrated V3–V4 dataset (Tracks 2 and 3) and (**B**) the V4 dataset (Track 1).

Differential abundance testing highlighted that the geographical origin significantly affects the auxiliary composition of the rhizosphere, revealing distinct taxonomic determinants in each national context (Figure 5). In the Chinese V3–V4 hypervariable profile (Figure 5A), environmental and non-enteric soil specialists significantly influenced regional niche differentiation. The obligate anaerobe *Holophaga*, carbohydrate-degrading specialist *Arachidicoccus*, and Gram-negative aerobic *Agriterribacter* exhibited the most substantial statistical variation across geographic coordinates (−log10(*p*-value) ~25) [26,27,28]. These were accompanied by additional important taxa such as SH-*PL14*, *Micropepsis*, *Chujaibacter*, *Taibaiella*, and *Fimbriimonas*. Other significant environmental factors with considerable regional variations included organic material-degrading *Sphingopyxis*, *Sphingorhabdus*, and *Chitinophaga* (−log10(*p*-value) ~22) [29,30,31] (Figure 5A).

Conversely, the Korean V4 hypervariable track (Figure 5B) revealed a distinctly different taxonomic group primarily comprising fermentative or highly adaptable root-associated soil organisms. *Ligilactobacillus* showed the greatest statistical variability among geographical regions (−log10(*p*-value) > 20), accompanied by significant regional abundance changes in *Escherichia-Shigella*, *Turicibacter*, *Romboutsia*, and *Bacteroides* (−log10(*p*-value) > 15). Importantly, the major functional nitrogen-cycling taxa displayed notable geographical differences; the chemolithoautotrophic nitrite-oxidizing genus *Nitrospira* and the nitrogen-fixing root nodule symbiont *Bradyrhizobium* exhibited highly significant regional variability in abundance (−log10(*p*-value) ~10) [32,33] (Figure 5B).

### 2.7. Ecological Trait Mapping Reveals Regional Differences in Putative Rhizosphere Functions

**Figure 6:**
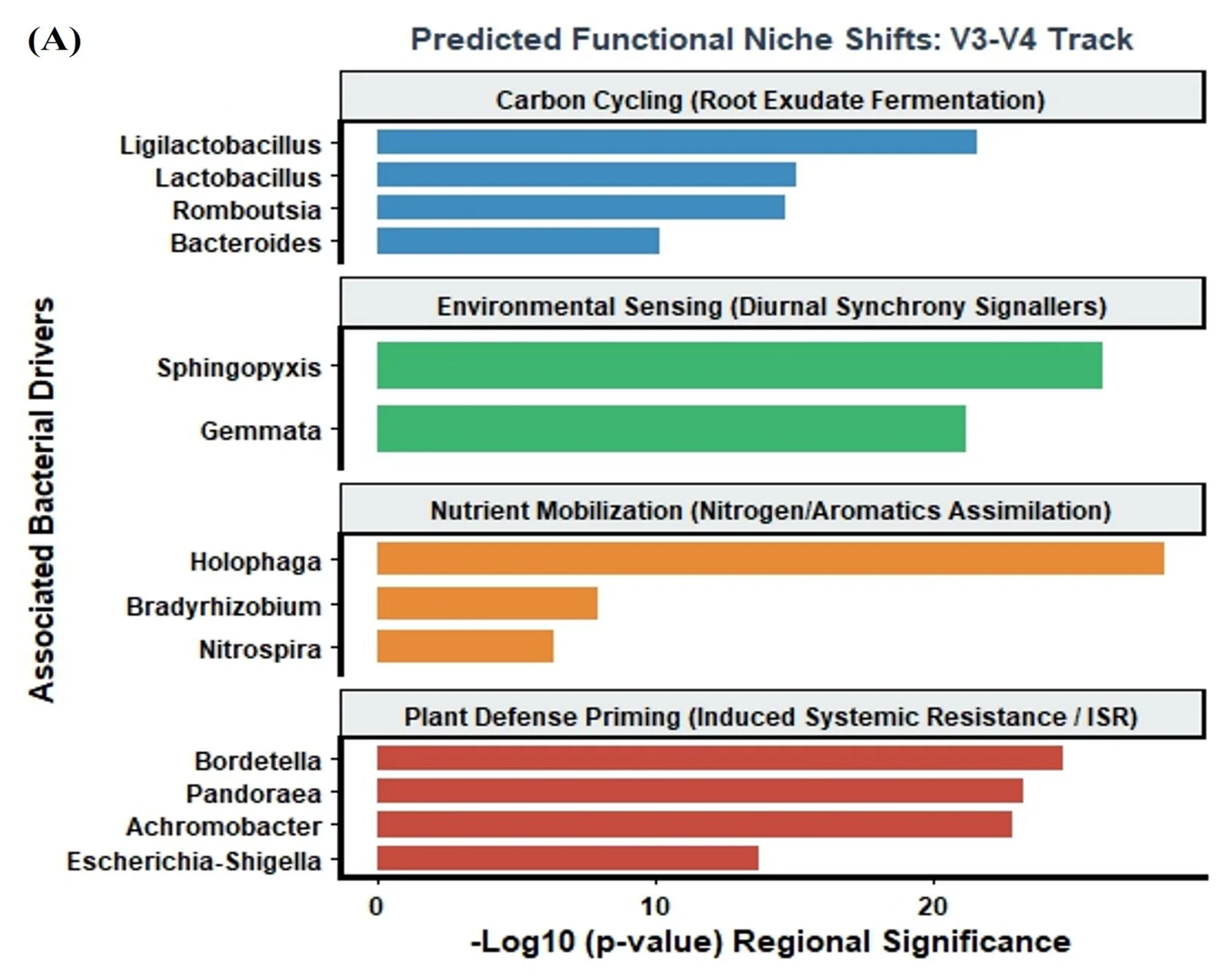

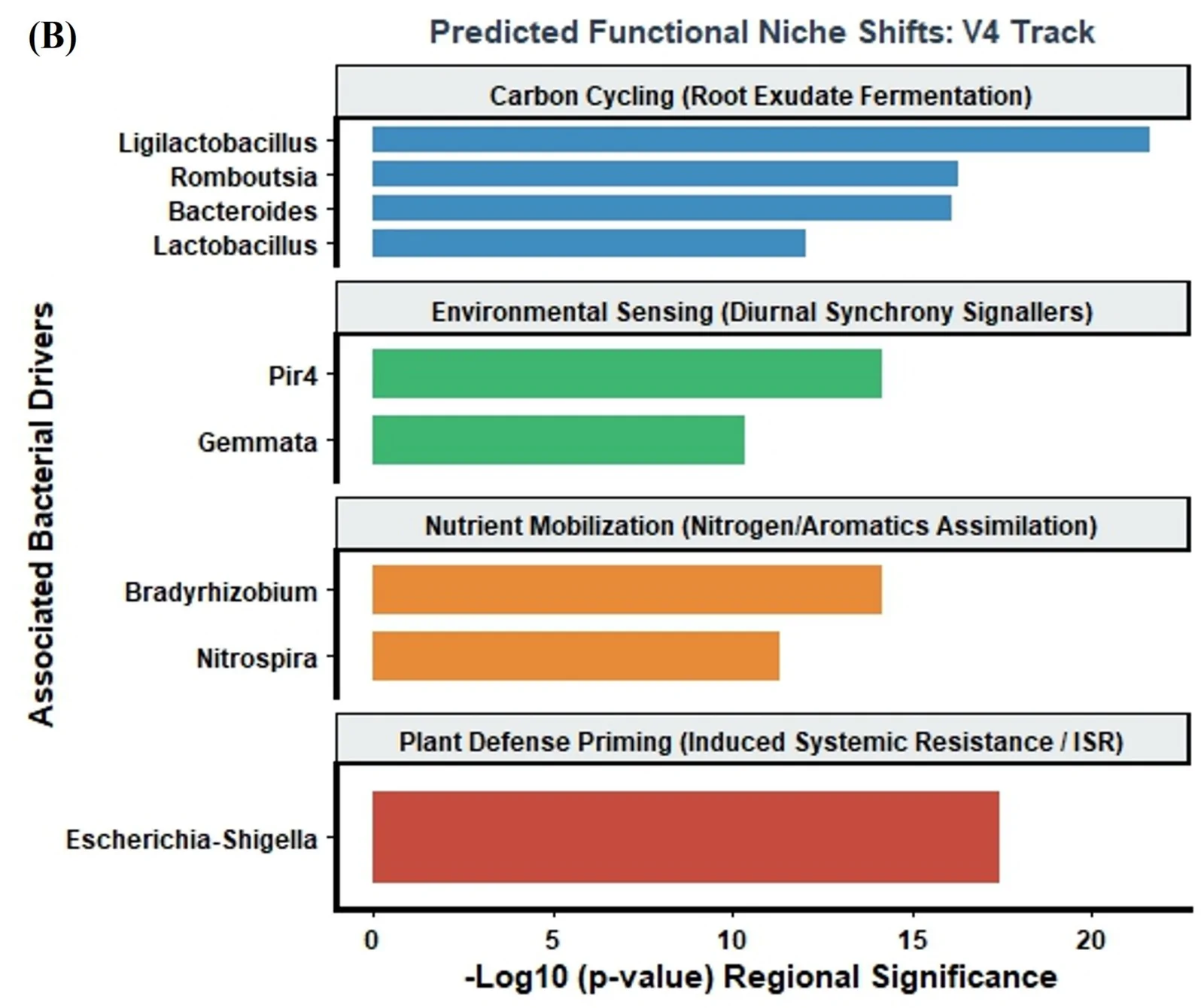
Ecological trait mapping across regional *Panax ginseng* rhizosphere samples. Four main eco-functional categories were identified from the differentially abundant bacterial genera (*p* < 0.05, Kruskal–Wallis test): carbon cycling (root exudate fermentation), environmental sensing (diurnal synchrony signaling), nutrient mobilization (assimilation of nitrogen and aromatic compounds), and plant defense priming (induced systemic resistance; ISR). The statistical significance of bacterial taxa linked to these functional categories in (**A**) the combined V3–V4 dataset and (**B**) the V4 dataset is shown by bars that show −log_10_(*p*-value).

Regional differences in bacterial genera were associated with their potential ecological functions in the rhizosphere by ecological trait mapping (Figure 6). In the Chinese V3–V4 dataset (Figure 6A), environmental sensing (diurnal synchronous signals) emerged as a key functional node, which was strongly supported by the prevalent regional differences driven by *Sphingopyxis* (−log10(*p*-value) > 26). Simultaneously, nutrient mobilization pathways exhibited considerable geographic diversity influenced by marked changes in *Holophaga*, indicating localized changes in the aromatic carbon turnover capacity of the rhizosphere across Chinese provinces (Figure 6A).

In the Korean V4 hypervariable dataset (Figure 6B), the functional landscape distinctly shifted towards metabolic interactions near the root. The carbon cycling (root exudate fermentation) network exhibited significant regional variations, driven by *Ligilactobacillus*, *Romboutsia*, and *Bacteroides* (−log10(*p*-value) > 16), indicating that the utilization of plant-origin low-molecular-weight exudates differed considerably across Korean domestic farms. Significantly, the plant defense priming (induced systemic resistance / ISR) module displayed a strong sensitivity to geographical origin, influenced by marked regional variations in *Escherichia-Shigella* (−log10(*p*-value) > 17) (Figure 6B). However, these attribute assignments do not directly show regional variations in microbial functional capacity or activity; they do offer an ecological context for interpreting the observed taxonomic patterns.

## 3. Discussion

The relatively stable Shannon diversity indices in different domestic and global microregions could provide valuable insights into the assembly processes of the *P. ginseng* holobionts (Figure 2). In ambient soil ecosystems, opportunistic environmental elements, such as seasonal temperature variations, varying nitrogen–phosphorus–potassium (NPK) ratios, and localized soil pH gradients, typically lead to significant changes in bacterial diversity and species uniformity [34,35,36]. However, the limited and overlapping vertical ranges observed across the 11 distinct Korean regions and most Chinese fields indicate fairly stable alpha-diversity patterns across the investigated locations, yet the causes of this stability cannot be discerned from the existing data.

This ecological balance suggests that *P. ginseng* may exert strong selective pressure on its adjacent rhizosphere environment, shaping the composition and functional capacity of the associated microbial populations [6]. The host plant functions as an ecological filter by continuously releasing targeted carbon-dense root exudates composed of organic acids, phenolic signals, and unique secondary metabolites [37,38]. This focused exudation could potentially restrict the growth of dominant opportunistic soil strains, preserving ideal baseline diversity [39]. Significant decreases in median diversity and wider distribution ranges observed in particular cohorts, such as Harbin and Tonghua, indicate the limits at which severe localized soil stress or long-term continuous cropping methods could surpass a plant’s ability to adapt. Through its effect on rhizosphere microbial assembly, *P. ginseng* plays a role in preserving distinct alpha diversity patterns and fostering the growth of functionally significant microbial communities throughout production areas [40].

By integrating the spatial configuration of the Bray–Curtis PCoA matrices (Figure 3) with the taxonomic classification of the 37 core bacteria (Supplementary Table S2), the ecological principles that influence *P. ginseng* holobionts were revealed. Our comprehensive data revealed that the rhizosphere microbiome of *P. ginseng* consists of a fairly stable core microbial community, with variations in accessory taxa that are specific to different regions. These patterns might indicate the combined influence of host-related and environmental factors, though the available data does not allow for differentiating their relative effects [6]. The overlap of confidence ellipses among Korean cultivation sites in the V4 matrix (Figure 3B), coupled with the relatively small percentage of variation due to geographic factors (R^2^ = 7.9%), suggests that rhizosphere microbial communities across Korea are fairly consistent, reflecting common host-related selection processes. In contrast, the Chinese provinces exhibited more pronounced regional differences (Figure 3A). As geographical origin was associated with BioProject identity, it was impossible to completely distinguish between study-specific and regional impacts. As a result, the PERMANOVA results are interpreted as patterns related to geography while acknowledging that the observed community differences may also have been influenced by study-specific factors.

The dominance of specific bacterial lineages among the 37 core taxa provides crucial insights into the unique ecology of the root zones (Supplementary Table S2). The consistent presence of Acidobacteriota and Gemmatimonadota across the included ginseng datasets suggests that these groups are stable constituents of the ginseng rhizosphere microbiome, potentially contributing to ecological functions under cultivation conditions [41]. The repeated presence of these oligotrophic, acid-tolerant phyla suggests a relatively stable baseline equilibrium within the rhizosphere holobiont [42,43,44,45]. These organisms have unique adaptations that might enable them to thrive and function in low-nutrient and low-pH environments, strengthening the root-soil connection throughout the host’s multiyear growth cycle [46].

From a biological perspective, the persistent presence of certain widely conserved genera, such as *Sphingomonas* and *Pseudolabrys*, in the cross-regional core has significant agronomic and defensive implications (Supplementary Table S2). *Sphingomonas* species are versatile root-colonizing bacteria that may enhance root development, pathogen resistance, production of phytohormones such as gibberellins, auxins, and indole acetic acid, and nitrogen cycling [37,47]. They may also contribute to the degradation and removal of inorganic pollutants from agricultural soils and increase plant resistance to drought, salinity, and heavy metal stress [48,49,50]. Concurrently, *Pseudolabrys* has been reported as a rhizobacterium that promotes plant growth, aids in plant development, breaks down hydrocarbon, and plays a significant role in the nitrogen cycle [51,52]. It has also been reported to have favorable lipid associations, which are adaptable signaling molecules that control numerous physiological processes in plants such as energy storage, stress response, protection against dehydration and pathogens, and the production of cellular membranes [53,54,55]. Additionally, the recurrent presence of *Gaiella* as a core taxon indicates its stable and widespread distribution across the studied groups [56,57,58] (Supplementary Table S2). Members of *Gaiella* have been reported to be involved in soil nitrogen cycling, particularly nitrate reduction and the decomposition of complex organic materials, and serve as vital functional collaborators despite vast geographic and national divides [59,60]. *Mycobacterium*, *Bryobacter*, and *Haliangium* could enhance plant health through various rhizosphere functions. These taxa are associated with nutrient and carbon cycling, organic matter decomposition, nitrogen transformation, pollutant degradation, and maintenance of a beneficial rhizosphere microenvironment, thereby potentially supporting plant growth and overall rhizosphere health [61,62,63,64,65]. A diverse bacterial community has been reported in taxonomic composition with Proteobacteria, Actinobacteriota, Acidobacteriota, Firmicutes, and Bacteroidota frequently observed in both the Chinese and Korean datasets. The variations in relative abundance at more specific taxonomic levels reveal significant diversity within rhizosphere communities. Nonetheless, variations between the two datasets must be viewed carefully due to disparities in geographic origin, BioProject structure, and sequencing area.

The identification of distinct microbial associations and anticipated functional pathways in the V3–V4 and V4 datasets emphasizes the potential impact of local environmental conditions on the composition and functional capabilities of the *P. ginseng* rhizosphere microbiome [5,6] (Figure 5 and Figure 6). The regional associations of *Holophaga Arachidicoccus*, and *Agriterribacter* in the Chinese V3–V4 profiles could indicate variations in local rhizosphere environmental factors, including soil pH, moisture, organic matter, and substrate availability, all of which are reported to affect *P. ginseng* rhizosphere microbial communities [66] (Figure 5A). *Holophaga* species are well-researched specialists that may contribute to metabolize complex aromatic compounds in nutrient-deficient terrestrial ecosystems [67]. The geographic differences may suggest that variations in local soil and rhizosphere environments could be associated with unique microbial functional profiles at different sampling locations, potentially affecting the decomposition of complex organic matter [68].

The alternative evolutionary group represented in the Korean V4 track, characterized by the presence of *Ligilactobacillus*, *Turicibacter*, and *Romboutsia* (Figure 5B), introduced a vibrant metabolic dimension to this regional distinction. These genera are highly effective in fermentation, rapidly metabolizing carbohydrates and producing organic acids [69,70]. These geographic variations may influence regional differences in rhizosphere conditions, possibly encompassing variations in root exudation linked to soil characteristics and climate factors. Nevertheless, the composition of root exudates was not assessed in this study, resulting in this being a potential ecological explanation rather than a confirmed mechanism [39].

The ecological trait within the *P. ginseng* rhizosphere may vary owing to regional differences in bacterial taxa and co-occurrence patterns (Figure 6). The consistent association between *Bradyrhizobium* and *Nitrospira* with nitrogen-related functions previously reported across both profiling tracks suggests a relatively conserved ecological association, and differences in taxa related to organic acid fermentation and environmental sensing may be attributed to regional variations in root-derived substrates and local environmental conditions [71,72]. The diverse ecological functions of rhizosphere bacteria have also been reported to be associated with the degradation of aromatic compounds and processes related to plant defense [40,73]. Overall, these results imply that certain taxonomic and co-occurrence patterns are common across regions, while others exhibit geographic differences.

### Limitations of the Study

Although this study provides a comprehensive synthesis of the P. ginseng rhizosphere microbiome across different growth regions, methodological and biological limitations should be considered when interpreting the results. The geographic distribution of the dataset was uneven, as it included one BioProject from Korea with 206 SRA samples and six BioProjects from China with 153 SRA samples. Microbial community comparisons may have been affected by differences in sequencing regions (V3–V4 versus V4), experimental methods, and study-specific batch effects. Therefore, variations between the V3–V4 and V4 datasets should be interpreted carefully, as the impacts of sequencing region, geographic origin, and BioProject could not be separately analyzed. Additionally, limited environmental metadata complicates the evaluation of factors related to soil and agricultural practices. Therefore, these regional trends should be interpreted with caution and further validated through standardized sampling across diverse geographic populations. These limitations in methods and sampling should also be acknowledged when interpreting trait-based analyses, which provide exploratory insights rather than definitive evidence of geographic differences in how microbes interact or their functional abilities.

## 4. Materials and Methods

### 4.1. Collection of Publicly Available Original 16S rRNA Amplicon Data

**Figure 7:**
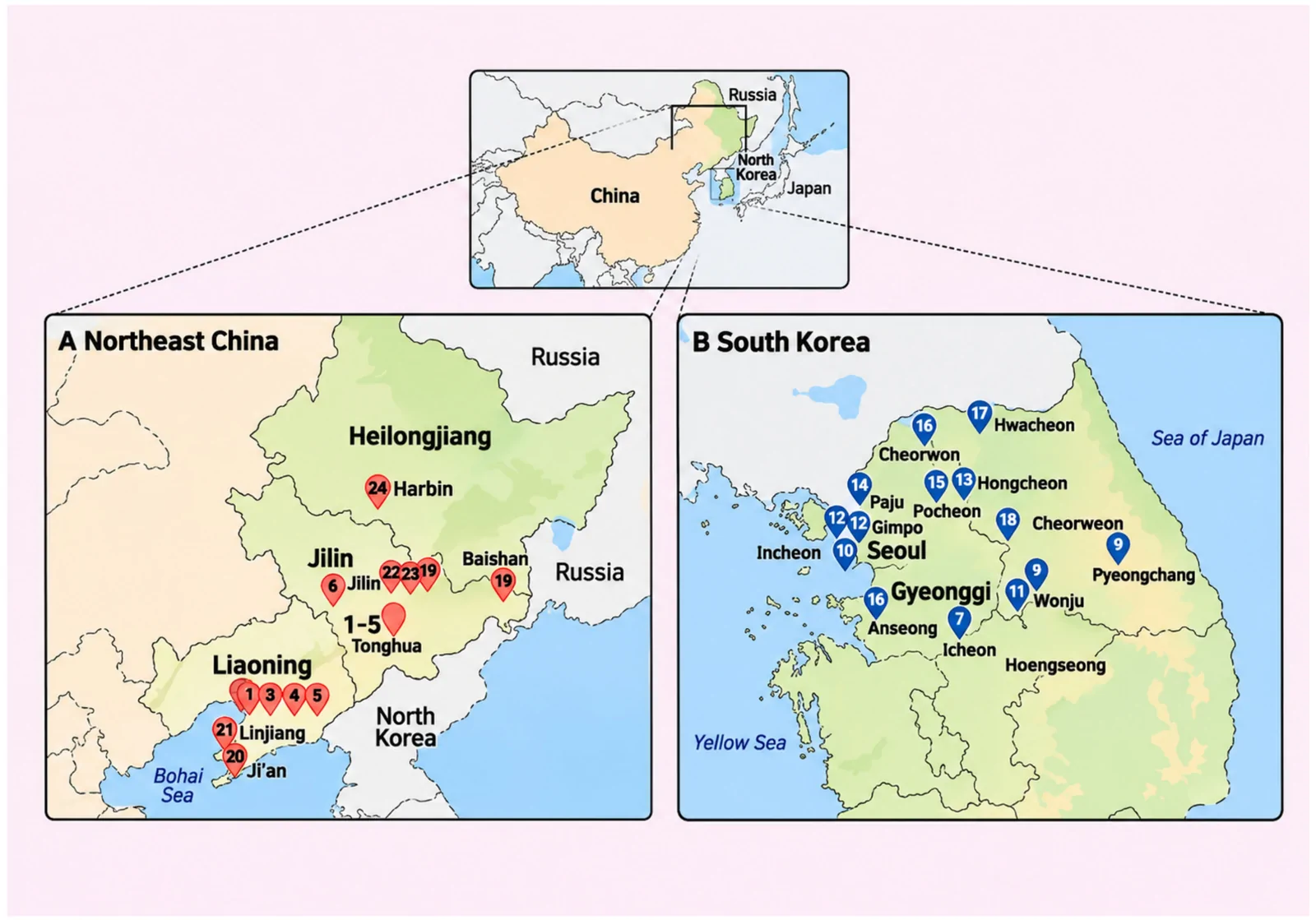
Geographic distribution of sampling sites featured in the *Panax ginseng* rhizosphere microbiome research across China and South Korea. Created with Biorender.com.

A comprehensive data-mining strategy was adopted to systematically map the bacterial microbiome of the *P. ginseng* rhizosphere, utilizing the NCBI BioProject database with the search term “(*Panax ginseng* rhizosphere)”, which initially yielded 21 potential matches. The selected studies were filtered based on the following rigorous inclusion criteria: (i) datasets must consist of bacterial 16S rDNA amplicon sequencing data exclusively from the Illumina platform (MiSeq, HiSeq, or NovaSeq); (ii) each experimental group must include at least five biological replicates for each experimental group; (iii) sample matrices must accurately reflect root-associated *P. ginseng* rhizosphere soil; and (iv) metadata must contain a confirmed geographical origin. These criteria narrowed the selection to seven eligible BioProjects, including six studies from China and one from South Korea (Table 1; Supplementary Table S3) (Figure 7). Using the SRA Toolkit, raw sequence data from these seven projects were retrieved and consolidated into a single meta-cohort, which included 359 unique SRA sample profiles of bacterial 16S rDNA sequences for further bioinformatic analysis. Not all datasets had consistently available to detailed information on DNA extraction, DNA quality, and library preparation, making it impossible to systematically assess any potential study-specific impacts.

To address significant batch effects, technological disparities, and variations in the experimental design of the multicohort microbiome integration, the combined sequencing dataset was organized into a three-track preprocessing framework. To enhance technical consistency across analyses and ensure alignment between single-end and paired-end sequencing methods, all datasets were processed using a standardized forward-read-only method. In this framework, Track 1 consisted solely of samples from the BioProject PRJNA707387, focusing on the 16S rRNA V4 hypervariable region and generating single-end reads on the Illumina MiSeq platform. Track 2 included V3–V4 amplicon datasets from BioProjects PRJNA795020, PRJNA681095, and PRJNA927237, which were generated using traditional sequencing platforms with continuous quality score distributions. Conversely, Track 3 included the V3–V4 datasets from BioProjects PRJNA704771, PRJNA701796, and PRJNA11191688, which were produced using more recent high-throughput sequencing technologies characterized by discretized (binned) quality-score profiles. This stratification facilitated platform-specific quality control along with error modeling and trimming methods, thus minimizing the technical variation associated with instruments before data integration [21]. Preprocessing specific to each track was utilized to address differences related to sequencing, while potential confounding factors such as sequencing region, geographic origin, and BioProject were taken into account when analyzing subsequent comparisons. Apart from preprocessing, significant statistical issues related to amplicon sequencing data, especially compositionality and the prevalence of zero counts, were addressed using subsequent analytical methods. Specifically, centered log-ratio (CLR) transformation was utilized to mitigate compositional constraints, and robust differential abundance techniques were applied to manage zero inflation and improve the reliability of cross-study analyses. To manage zero inflation, a pseudocount equivalent to half of the minimum non-zero detection limit was applied before transformation. The natural logarithm of each taxon’s abundance in relation to the sample-specific geometric mean was used to determine CLR values. In addition to stabilizing variance, reducing skew from highly dominant taxa without eliminating extreme results, and enabling reliable downstream differential abundance testing across heterogeneous multi-study cohorts, this transformation converts data from simplex to Euclidean space [74].

### 4.2. Sequence Quality Trimming, DADA2 Processing, and Amplicon Sequence Variants (ASVs) Inference

All analyses were performed in R (version 4.6.0) using the DADA2 (Divisive Amplicon Denoising Algorithm) package (version 1.40.0) to extract high-resolution ASVs (Amplicon sequence variants) from raw sequencing data. DADA2 was applied for quality filtering, denoising, error modeling, and removal of chimeric sequences. To manage the differences in the targeted 16S rRNA hypervariable regions and the error profiles specific to different sequencing platforms, the seven BioProjects were categorized into three distinct analytical tracks for separate processing. Taxonomic classifications were assigned to the resulting ASVs using the SILVA reference database (version 138.2).

Quality assessment was initially performed on the forward reads to establish consistent trimming standards across all datasets. The FilterAndTrim function in DADA2 was used for quality filtering and trimming, applying a consistent truncation length of 200 bp to eliminate low-quality terminal regions while maintaining consistent read lengths for subsequent analyses. A strict upper limit on the expected error (maxEE = 2) was applied to filter reads with high cumulative sequencing errors. During pre-processing, reads containing ambiguous nucleotides (maxN = 0) and those matching standard PhiX control sequences were discarded.

Following quality control, the sequencing error models were independently assessed for each analytical track using the LearnErrors algorithm to address track-specific error traits. These error profiles were then integrated into a data-denoising process to derive high-resolution ASVs, allowing for the differentiation of true biological sequence variants from sequencing errors with single-nucleotide accuracy. To ensure methodological consistency across analysis with different library configurations, a forward-read-only processing strategy was utilized, and paired-read merging was omitted. Finally, chimeric sequences were identified and eliminated de novo using a consensus-based method in the removeBimeraDenovo function.

The rarecurve() function in the vegan package in R was used to create individual rarefaction curves at 100-read intervals for every sequencing track. Sequencing adequacy was evaluated by plotting observed ASV richness against sequencing depth. There were 359 samples in the combined dataset (Track 1, n = 206; Track 2, n = 90; Track 3, n = 63). Sample and BioProject summarized the raw, filtered, denoised, and final non-chimeric reads. The formulas for filtering and final retention were filtered reads/raw reads × 100 and final non-chimeric reads/raw reads × 100, respectively. When pre-chimera counts could be recovered, denoised reads minus final non-chimeric reads were used to calculate the number of chimeric reads eliminated.

ASV tables were filtered to eliminate mitochondria, chloroplasts, and non-bacterial taxa. To take into consideration variations in sequencing depth, ASV read counts were transformed into relative abundances. While alpha diversity was computed using rarefied tables, beta diversity, differential abundance, core microbiome, and network analyses used filtered relative-abundance data.

### 4.3. Taxonomic Assignment and Phyloseq Integration

Taxonomically classified ASVs were organized using the RDP Naive Bayesian classifier integrated into DADA2 [75] and cross-referenced using the SILVA database (v138.2) [76], ensuring a minimum bootstrap confidence of 80% for assignments. Taxonomic annotations were merged with sample metadata and ASV abundance tables to create cohesive phyloseq objects (phyloseq _v3v4_clean.rds and phyloseq _v4_clean.rds) (Supplementary Table S4). These objects provide a cohesive data framework for subsequent analyses, including community diversity evaluation, abundance variation testing, ecological functional annotation, and microbial co-occurrence network identification.

### 4.4. Alpha Diversity Profiling and Homeostatic Assessments

Alpha diversity was calculated to characterize the ecological complexity and within-sample diversity of the rhizosphere microbiomes of *P. ginseng*. Analyses were performed at the ASV level using the estimate_richness function in the phyloseq R package. The Shannon diversity index (H′) was used as the primary diversity metric, as it accounts for both taxonomic richness and abundance evenness.

### 4.5. Beta Diversity Modeling and Functional Core Integration

Patterns of beta diversity in the rhizosphere microbiomes of *P. ginseng* were analyzed using Bray–Curtis dissimilarity and depicted using principal coordinates analysis (PCoA). To normalize variations in sequencing depth, ASV abundances were converted into relative abundances before constructing the distance matrix. The geographic variation in community composition was statistically evaluated using PERMANOVA (adonis2, 999 permutations), with geographic location as the grouping variable. This analysis yielded estimates of statistical significance (*p*-value), effect size (R^2^), and pseudo-F values, which illustrated the degree of community differentiation across the cultivation regions.

### 4.6. Cross-Study Core Microbiome Identification and Taxonomic Consolidation

The core microbiome was analyzed using independently processed phyloseq datasets to identify taxa consistently associated with *P. ginseng* rhizosphere across various geographic regions and sequencing platforms. Separate analyses were performed for each sequencing track to minimize bias in the classification related to the primers and platforms. Before identifying the core microbiome, ASV counts were aggregated at the genus level using tax _glom and converted to relative abundances to adjust for variations in sequencing depth across samples and BioProjects.

Core taxa were identified using the microbiome package based on the prevalence–abundance model. A genus was classified as part of the core microbiome if it appeared in ≥80% of samples (prevalence ≥ 0.80) and had a minimum relative abundance of 0.1% (abundance ≥ 0.001) [77,78,79]. These criteria facilitated the identification of prevalent and consistently observed core taxa within the *P. ginseng* rhizosphere microbiome.

### 4.7. Taxonomic Structure and Relative Abundance Analysis

The taxonomic relative abundances were outlined at the levels of phylum, class, order, and genus. For every sample, the relative abundance of each taxon was calculated by assessing the proportion of sequence reads attributed to that taxon in relation to the total classified reads within the respective sample. The most prevalent taxa were shown separately in the taxonomic composition graphs, whereas less prevalent taxa were grouped into an “Others” category exclusively for visualization. The top 10 taxa were shown at the phylum, class, and order levels, while the leading 15 taxa were shown at the genus level.

### 4.8. Differential Taxonomic Abundance Profiling

To identify the bacterial genera influencing geographic differences in the *P. ginseng* rhizosphere microbiome, differential abundance analyses were performed using a non-parametric statistical method in R. The abundance of ASVs was aggregated at the genus level, and taxa with low prevalence were excluded to minimize the background noise and potential technical errors. Genera were included in the analysis only if they showed a minimum of five reads in at least 10% of the samples. Variations in genus abundance across different geographic areas were evaluated using the Kruskal–Wallis test, and the resulting *p*-values were adjusted for multiple comparisons using the Benjamini–Hochberg false discovery rate (FDR) approach. Genera with noteworthy FDR-adjusted values were considered potential microbial contributors to regional niche differentiation.

### 4.9. Ecological Trait Mapping

Regional variations in bacterial genera were interpreted in terms of their potential ecological roles using an ecological trait mapping approach. Bacterial genera exhibiting statistically significant regional variations were assigned to fundamental ecological operational modules according to their reported physiological and metabolic characteristics in terrestrial ecosystems. The functional networks were characterized by carbon cycling (fermentation of root exudates), nutrient mobilization (assimilation of nitrogen/aromatics), environmental sensing (signals of diurnal synchrony), and plant defense priming (induced systemic resistance/ISR). The five bacterial genera with the greatest divergence within each functional operational module were dynamically isolated according to their relative abundances using Kruskal–Wallis *p*-values. These assignments offer a descriptive analysis of the potential ecological functions of the identified genera and do not reflect direct assessments of microbial functional ability or activity.

## 5. Conclusions

To sum up, this cross-regional amplicon sequencing data analysis presents an extensive overview of the ginseng rhizosphere microbiome, exposing hypervariable profiling tracks and divergent taxonomic and functional shifts across regional microenvironments. In particular, regional beta diversity ordinations (PCoA) showed strong spatial structure between Chinese and Korean rhizosphere ecosystems after managing for study batch effects through BioProject strata. Taxonomic analysis extracted essential bacterial genera influencing regional differences, while abundance profiles underscored significant regulators of microbial community structure. Additionally, the systemic synthesis of read-retention tracking and metadata pipelines created a strict framework for cross-study comparability, proving that multi-dataset integration can consistently reveal microbial community patterns without the limitations of conventional wet-lab techniques. The findings showed that geographic origin plays a crucial role in determining the composition of rhizosphere communities, affecting their taxonomic structure and functional capabilities, beyond the scope of individual studies.

A comparative analysis of the two sequencing tracks revealed distinct patterns in bacterial community structure and the distribution of regionally associated taxa. Significantly, geographic variations in key nutrient-cycling genera, such as *Nitrospira* and *Bradyrhizobium*, along with plant-associated groups such as *Escherichia-Shigella* and *Achromobacter*, indicate that regional variations in the rhizosphere microbiome may be linked to regional variations in rhizosphere microbial populations; however, experimental validation is necessary to confirm these potential functionalities. Collectively, these findings establish a basis for future microbiome-based strategies in medicinal crop production, including targeted microbial management, synthetic community development, and localized precision agriculture techniques. However, the variations observed in microbial community structure and predicted ecological traits must be interpreted with care due to the overlapping effects of geographic source, BioProject identification, sequencing region, and methodological aspects. Consequently, trait-based analyses are exploratory and indicate patterns at the dataset level rather than verified geographic impacts.

## Figures and Tables

**Table 1:** An overview of the seven BioProjects and sequencing datasets that were included in the *Panax ginseng* rhizosphere microbiome integrated reanalysis.

No	Bioprojcet	Region	Number Samples (n)	Collection Time	Primers	Total Reads	Layout	Platform	Country
1	PRJNA795020	V3-V4	36	Not found	Not found	Not found	Paired	Illumina miseq	China
2	PRJNA704771	V3-V4	30	July 2020	341F (5′-ACTCCTACGGGAGGCAGCAG-3′) 806R (5′-GGACTACHVGGGTWTCTAAT-3′)	Not found	Paired	Illumina novaseq600	China
3	PRJNA707387	V4	206	August 2020	Not found	Not Found	Single	Illumina miseq	Korea
4	PRJNA701796	V3-V4	18	August 2018	341F (5′-ACTCCTACGGGAGGCAGCAG-3′) 806R (5′-GGACTACHVGGGTWTCTAAT-3′)	1,135,354	Paired	Illumina hiseq x ten	China
5	PRJNA681095	V3-V4	36	June 2017	338F(5′-ACTCCTACGGGAGGCAGCAG-3′) 806R(5′-GGACTACHVGGGTWTCTAAT-3′)	1,362,294	Single	Illumina miseq	China
6	PRJNA927237	V3-V4	18	July 2020	338F(5′-ACTCCTACGGGAGGCAGCAG-3′) 806R(5′-GGACTACHVGGGTWTCTAAT-3′)	1,429,596	Paired	Illumina hiseq2000	China
7	PRJNA1191688	V3-V4	15	September 2022	341F (5′-ACTCCTACGGGAGGCAGCAG-3′) 806R (5′-GGACTACHVGGGTWTCTAAT-3′)	6,011,861	Paired	Illumina novaseq600	China

## Data Availability

All data obtained in this study are included in the article. Please contact the corresponding author for further information. All reproducible R scripts and data processing workflows generated during this study are publicly accessible in the Figshare repository (https://doi.org/10.6084/m9.figshare.33339468).

## Supplementary Materials

The following supporting information can be downloaded at: https://www.mdpi.com/article/10.3390/plants15213473/s1.

## Abbreviations

The following abbreviations are used in this manuscript:

ASV	Amplicon Sequence Variant
CLR	Centered Log-Ratio
FDR	False Discovery Rate
ISR	Induced Systemic Resistance
NGS	Next-Generation Sequencing
NPK	Nitrogen–Phosphorus–Potassium
PERMANOVA	Permutational Multivariate Analysis of Variance
PCoA	Principal Coordinates Analysis
RDP	Ribosomal Database Project
SRA	Sequence Read Archive
V3–V4/V4	Hypervariable Regions of 16S rRNA Gene Sequencing
