# Supplementary material for: Cross-Regional Reanalysis of 16S rDNA Amplicon Sequence Data Reveals Core Taxa and Regional Modular Dynamics in the Panax ginseng Rhizosphere Bacterial Community
Source: Plants (Basel). 2026 Nov 6;15(21):3473. doi: 10.3390/plants15213473 (PMC13765625; doi:10.3390/plants15213473)
Supplement: Supplementary file 1 [file plants-15-03473-s001.zip › Supplementary Table S1.pdf]

Supplementary Table S1: Read-retention summary for each BioProject.

BioProject	Processing_track	Region	Samples_n	Raw_reads	Filtered_reads	Denoised_reads	Chimeric_reads_removed	Final_non_chimeric_reads	Filtering_retention_percent	Final_retention_percent
PRJNA1191688	Track 3	V3-V4	15	1248789	1079025	866471	0	866471	86.41	69.38
PRJNA681095	Track 2	V3-V4	36	1362294	1327446	1240131	7168	1232963	97.44	90.51
PRJNA701796	Track 3	V3-V4	18	959677	943474	722303	0	722303	98.31	75.27
PRJNA704771	Track 3	V3-V4	30	2669770	2241959	2060616	0	2060616	83.98	77.18
PRJNA707387	Track 1	V4	206	6491486	6096896	5329937	194912	5135025	93.92	79.1
PRJNA795020	Track 2	V3-V4	36	1395500	1247560	1087790	83339	1004451	89.4	71.98
PRJNA927237	Track 2	V3-V4	18	988044	925773	808274	27635	780639	93.7	79.01
