# Supplementary material for: Cross-Regional Reanalysis of 16S rDNA Amplicon Sequence Data Reveals Core Taxa and Regional Modular Dynamics in the Panax ginseng Rhizosphere Bacterial Community
Source: Plants (Basel). 2026 Nov 6;15(21):3473. doi: 10.3390/plants15213473 (PMC13765625; doi:10.3390/plants15213473)
Supplement: Supplementary file 1 [file plants-15-03473-s001.zip › Supplementary Table S2.pdf]

Supplementary Table S2: Core bacterial microbiome comprising 37 taxa in the Panax ginseng rhizosphere samples

**Mean Relative Abundance (%)**	**Phylum**	**Class**	**Order**	**Family**	**Genus**
2.385	Pseudomonadota	Alphaproteobacteria	Hyphomicrobiales	Xanthobacteraceae	*Unclassified Xanthobacteraceae*
2.043	Gemmatimonadota	Gemmatimonadia	Gemmatimonadales	Gemmatimonadaceae	*Unclassified Gemmatimonadaceae*
1.955	Acidobacteriota	Vicinamibacteria	Vicinamibacterales	Incertae Sedis	*Unclassified Bacteria*
1.828	Actinomycetota	Thermoleophilia	Gaiellales	Gaiellaceae	*Gaiella*
1.027	Actinomycetota	Acidimicrobiia	Acidimicrobiales	Acidimicrobiaceae	*Acidiferrimicrobium*
0.919	Actinomycetota	Actinobacteria	Mycobacteriales	Mycobacteriaceae	*Mycobacterium*
0.471	Chloroflexota	TK10	Incertae Sedis	Incertae Sedis	*Unclassified Bacteria*
4.985	Acidobacteriota	Acidobacteriae	Terriglobales	Incertae Sedis	*Unclassified Bacteria*
3.24	Acidobacteriota	Vicinamibacteria	Vicinamibacterales	Incertae Sedis	*Unclassified Bacteria*
3.089	Gemmatimonadota	Gemmatimonadia	Gemmatimonadales	Gemmatimonadaceae	*Unclassified Gemmatimonadaceae*
1.909	Pseudomonadota	Alphaproteobacteria	Hyphomicrobiales	Xanthobacteraceae	*Pseudolabrys*
1.895	Pseudomonadota	Alphaproteobacteria	Sphingomonadales	Sphingomonadaceae	*Sphingomonas*
1.707	Pseudomonadota	Gammaproteobacteria	Burkholderiales	SC-I-84	*Unclassified SC-I-84*
1.523	Actinomycetota	Thermoleophilia	Gaiellales	Incertae Sedis	*Unclassified Bacteria*
1.456	Acidobacteriota	Acidobacteriae	Bryobacterales	Bryobacteraceae	*Bryobacter*
1.422	Chloroflexota	KD4-96	Incertae Sedis	Incertae Sedis	*Unclassified Bacteria*
1.247	Pseudomonadota	Gammaproteobacteria	Gammaproteobacteria Incertae Sedis	Unknown Family	*Acidibacter*
1.197	Acidobacteriota	Holophagae	Subgroup 7	Incertae Sedis	*Unclassified Bacteria*
1.16	Actinomycetota	Thermoleophilia	Gaiellales	Gaiellaceae	*Gaiella*
1.086	Bacillota	Clostridia	Clostridiales	Clostridiaceae	*Clostridium*
1.065	Chloroflexota	Ktedonobacteria	C0119	Incertae Sedis	*Unclassified Bacteria*
0.953	Gemmatimo	Gemmatimon	Gemmatimon	Gemmatimon	*Gemmatimonas*

	nadota	adia	adales	adaceae	
0.897	Verrucomicrobiota	Verrucomicrobiia	Pedosphaerales	Pedosphaeraceae	*Unclassified Pedosphaeraceae*
0.858	Verrucomicrobiota	Verrucomicrobiia	Chthoniobacterales	Chthoniobacteraceae	*Candidatus*
0.708	Actinomycetota	Actinobacteria	Mycobacteriales	Mycobacteriaceae	*Mycobacterium*
0.68	Actinomycetota	Thermoleophilia	Solirubrobacterales	67-14	*Unclassified 67-14*
0.675	Nitrospirota	Nitrospiria	Nitrospirales	Nitrospiraceae	*Nitrospira*
0.617	Planctomycetota	Planctomycetes	Gemmatales	Gemmataceae	*Unclassified Gemmataceae*
0.613	Planctomycetota	Phycisphaerae	Tepidisphaerales	WD2101 soil group	*Unclassified WD2101 soil group*
0.57	Planctomycetota	Planctomycetes	Pirellulales	Pirellulaceae	*Unclassified Pirellulaceae*
0.563	Methylomirabilota	Methylomirabilia	Rokubacteriales	Incertae Sedis	*Unclassified Bacteria*
0.485	Chloroflexota	TK10	Incertae Sedis	Incertae Sedis	*Unclassified Bacteria*
0.466	NA	NA	NA	NA	*Unclassified Bacteria*
0.462	Planctomycetota	Planctomycetes	Gemmatales	Gemmataceae	*Gemmata*
0.438	Myxococcota	Polyangiia	Haliangiales	Haliangiaceae	*Unclassified Haliangiaceae*
0.425	Pseudomonadota	Alphaproteobacteria	Reyranellales	Reyranellaceae	*Reyranella*
0.422	Planctomycetota	Planctomycetes	Planctomycetales	Incertae Sedis	*Unclassified Bacteria*
